# Identifying Breeding Priorities for Blueberry Flavor Using Biochemical, Sensory, and Genotype by Environment Analyses

**DOI:** 10.1371/journal.pone.0138494

**Published:** 2015-09-17

**Authors:** Jessica L. Gilbert, Matthew J. Guthart, Salvador A. Gezan, Melissa Pisaroglo de Carvalho, Michael L. Schwieterman, Thomas A. Colquhoun, Linda M. Bartoshuk, Charles A. Sims, David G. Clark, James W. Olmstead

**Affiliations:** 1 Plant Innovation Center, Institute of Food and Agricultural Sciences, University of Florida, Gainesville, Florida, United States of America; 2 Horticultural Sciences Department, Institute of Food and Agricultural Sciences, University of Florida, Gainesville, Florida, United States of America; 3 School of Forest Resources and Conservation, Institute of Food and Agricultural Sciences, University of Florida, Gainesville, Florida, United States of America; 4 Department of Environmental Horticulture, Institute of Food and Agricultural Sciences, University of Florida, Gainesville, Florida, United States of America; 5 Food Science and Human Nutrition, Institute of Food and Agricultural Sciences, University of Florida, Gainesville, Florida, United States of America; Fresno, UNITED STATES

## Abstract

Breeding for a subjective goal such as flavor is challenging, as many blueberry cultivars are grown worldwide, and identifying breeding targets relating to blueberry flavor biochemistry that have a high degree of genetic control and low environmental variability are priorities. A variety of biochemical compounds and physical characters induce the sensory responses of taste, olfaction, and somatosensation, all of which interact to create what is perceived flavor. The goal of this study was to identify the flavor compounds with a larger genetic versus environmental component regulating their expression over an array of cultivars, locations, and years. Over the course of three years, consumer panelists rated overall liking, texture, sweetness, sourness, and flavor intensity of 19 southern highbush blueberry (*Vaccinium corymbosum* hybrids) genotypes in 30 sensory panels. Significant positive correlations to overall liking of blueberry fruit (*P*<0.001) were found with sweetness (R^2^ = 0.70), texture (R^2^ = 0.68), and flavor (R^2^ = 0.63). Sourness had a significantly negative relationship with overall liking (R^2^ = 0.55). The relationship between flavor and texture liking was also linear (R^2^ = 0.73, *P*<0.0001) demonstrating interaction between olfaction and somatosensation. Partial least squares analysis was used to identify sugars, acids, and volatile compounds contributing to liking and sensory intensities, and revealed strong effects of fructose, pH, and several volatile compounds upon all sensory parameters measured. To assess the feasibility of breeding for flavor components, a three year study was conducted to compare genetic and environmental influences on flavor biochemistry. Panelists could discern genotypic variation in blueberry sensory components, and many of the compounds affecting consumer favor of blueberries, such as fructose, pH, β-caryophyllene oxide and 2-heptanone, were sufficiently genetically controlled that allocating resources for their breeding is worthwhile.

## Introduction

United States blueberry (*Vaccinium* spp.) consumption has increased fifteen-fold in the past two decades, from 27 to 429 million pounds between 1990 and 2013 [1]. Additionally, growth in consumption abroad has been fueled by the development of new markets, with demand now strong in Europe and Asia [2]. North America produces the most blueberries worldwide, but growth of global production has accelerated as breeding efforts have allowed the expansion of blueberry production into new growing areas, such as low chill regions and the Southern hemisphere. A previous psychophysical study conducted to identify blueberry consumer preferences suggested that consumers were most likely to purchase sweet berries with intense blueberry flavor [3]. However, identifying consumer blueberry preferences to determine what consumers desire in an ideal blueberry is only the first step in creating better products. Breeding for a subjective goal such as flavor is challenging, as many blueberry cultivars are grown worldwide, and identifying breeding targets relating to blueberry flavor biochemistry that have a high degree of genetic control and low environmental variability are priorities.

A variety of biochemical compounds and physical characters induce the sensory responses of taste, olfaction, and somatosensation, all of which interact to create what is perceived flavor. The primary tastes are sweet, sour, salty, bitter, and umami [4,5]. Olfaction is the sense mediated by a complex network of olfactory receptors; it dictates smell and flavor. Smell, or orthonasal olfaction, is the sensory perception of volatile compounds from the air through the nose. Flavor is the result of the same compounds when bound by the same receptors after being pushed up behind the palette (e.g. during mastication), called retronasal olfaction [6]. Volatiles are low-molecular weight lipophilic metabolites that come from various biosynthetic pathways. Thousands of these compounds contributing to aroma profiles of fruits have been identified in literature, some of which may be classified as spicy, flowery, fruity, resinous, balsamic, burnt, and foul [5,7]. These compounds may contribute to characteristic blueberry aromas, such as fruity, floral, peachy, or grassy-green [8], although these compounds may produce different sensations when perceived ortho- or retronasally [9]. Somatosensations are derived from the tactile, thermal, and irritation qualities. The three sensory modalities of taste, flavor, and somatosensation are mediated by nerve signals to the brain, in regions of overlap and specificity, and can act synergistically or suppressively when compounds interact structurally, chemically, or cognitively [4].

The biochemical components in fruit that contribute to flavor and taste are subject to the effects of blueberry genetics of different cultivars (G), production environment (E), and G×E interactions [10,11]. In breeding fruit for quality traits such as flavor, consistent performance of a cultivar between growing locations and seasons is desirable. Therefore, identifying compounds whose production is more regulated by genotype than environment or G×E interaction effects is preferable for breeders, who are unable to control E or G×E. Soluble solids content (SSC) and titratable acidity (TA) vary between *Vaccinium* species, cultivars, and years [12–15]. Similarly, volatile levels have been shown to vary between *Vaccinium* species, cultivars, locations, progression through fruiting season, and storage conditions [15–19]. It appears that the same volatile compound may exhibit different gradations of environmental variation in different genetic backgrounds, which sets the stage for a broader survey of G×E effects on sugars, acids and volatiles in the blueberry germplasm [15]. Environment represents the fruit variation resulting from changes in conditions during the course of a harvest season, including variables such as temperature, precipitation, crop load, and irrigation. For example, changes in blueberry (*V*. *corymbosum* L.) volatile components have been attributed to dosages of UV-B radiation and discrete light wavelengths [20,21].

In an effort to better understand the genetic components of fruit quality, southern highbush blueberry fruit subject to these numerous, uncontrollable environmental parameters were sampled to identify compounds that are important to the sensory experience, and would be worth allocating resources to target in breeding. The end goal was to identify sugar, acid, and volatile compounds with a larger genetic versus environmental variation over an array of cultivars, locations, and years. To accomplish this, 19 blueberry cultivars were assayed for biochemical variation and evaluated in 30 sensory panels over the course of three years. Panelists rated fruit quality to create associations between the sensory experience and blueberry biochemistry. Six blueberry genotypes were selected for G×E analysis, allowing for comparison of flavor biochemistry of cultivars amongst three locations with different latitudes, weather characteristics, and management practices.

## Results and Discussion

Over three seasons, 19 blueberry genotypes were assayed for overall liking, texture liking, perceived sweetness, sourness, and flavor intensities by 217 panelists in 30 sensory panels, and for the biochemical measures of sucrose, glucose, fructose, soluble solids content (SSC), titratable acidity (TA), pH, and 52 volatiles (S2 Table). A PLS model was constructed to determine the most relevant biochemical effectors of liking, intensity of sweetness, sourness, and blueberry flavor to be used for breeding selection. Six of these 19 genotypes (‘Emerald,’ ‘Endura,’ ‘Farthing,’ ‘Meadowlark,’ ‘Primadonna,’ and ‘Scintilla’) were sampled three times a season for three years in three locations to quantitate the biochemical variation due to environmental effects, and whether these differences were perceivable by humans.

### Blueberry genotypes vary significantly in scores for liking and perceived sensory intensities

**Fig 1:**
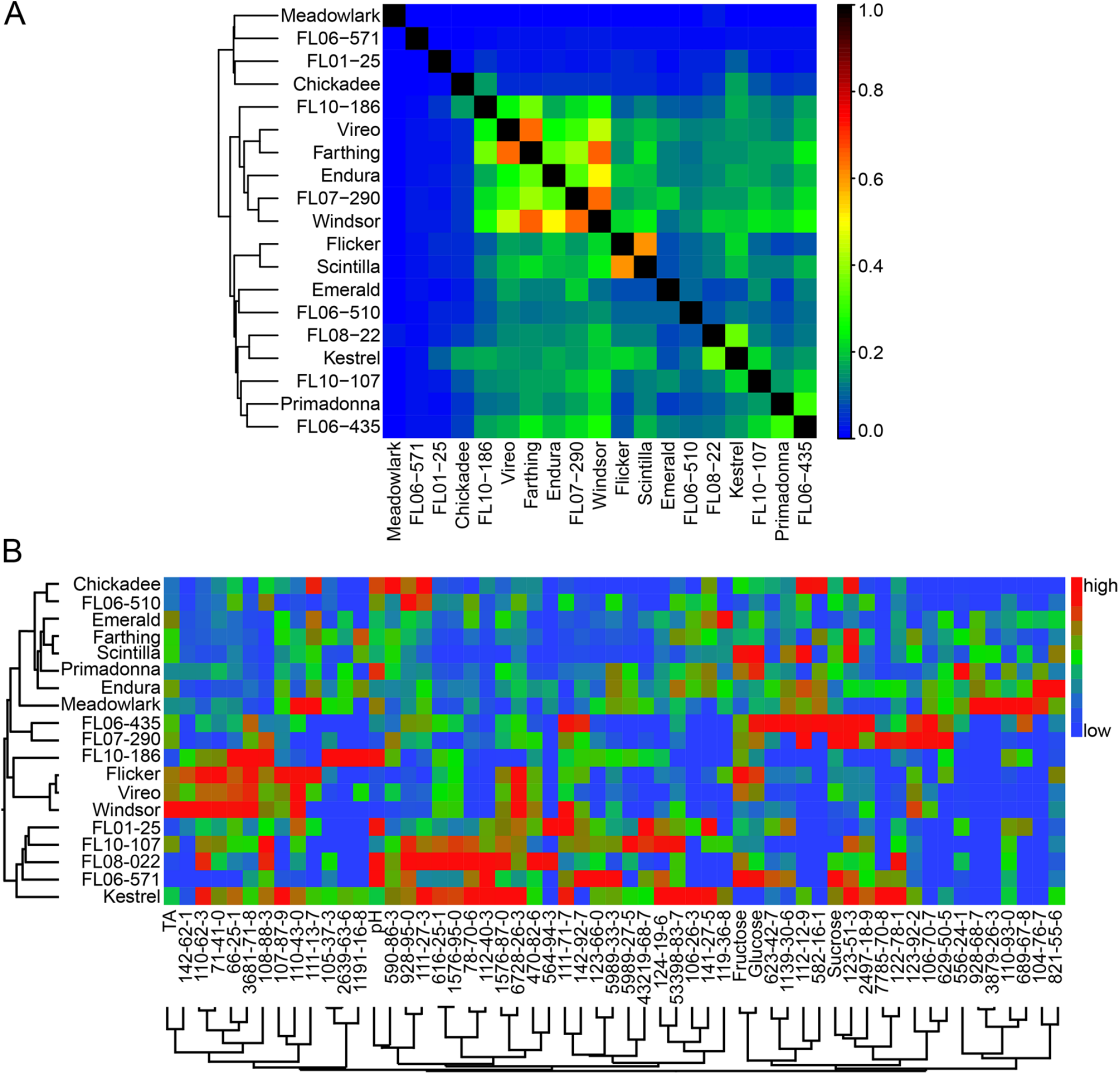
Genotypic relatedness based on pedigree and flavor biochemistry. (A) Relationships between genotypes sampled in this study, based on pedigree information (0 = no relation, to 1 = high relationship) with a hierarchical cluster analysis. The hierarchy and distance of the dendrogram indicates relatedness of genotypes. (B) Two-way ward cluster analysis of blueberry genotypes (left) and biochemical measures (bottom). High values are represented as red, average as green, low as blue. The hierarchy and distance of the dendrograms indicate relatedness of genotypes based on metabolite profiles.

Genotypic relatedness based on pedigree and biochemical profiles were compared using hierarchical cluster analyses (Fig 1). The dendrograms between the two analyses do not pair the same genotypes together in any instance, demonstrating the diversity of biochemistry that exists regardless of genotypic relatedness. This is likely due to the highly heterozygous nature of this autotetraploid species that extends beyond pedigree information. A cluster analysis based on molecular genetic relatedness between these individuals may show more similarity to biochemical clusters, and may be considered in future investigations.

**Fig 2:**
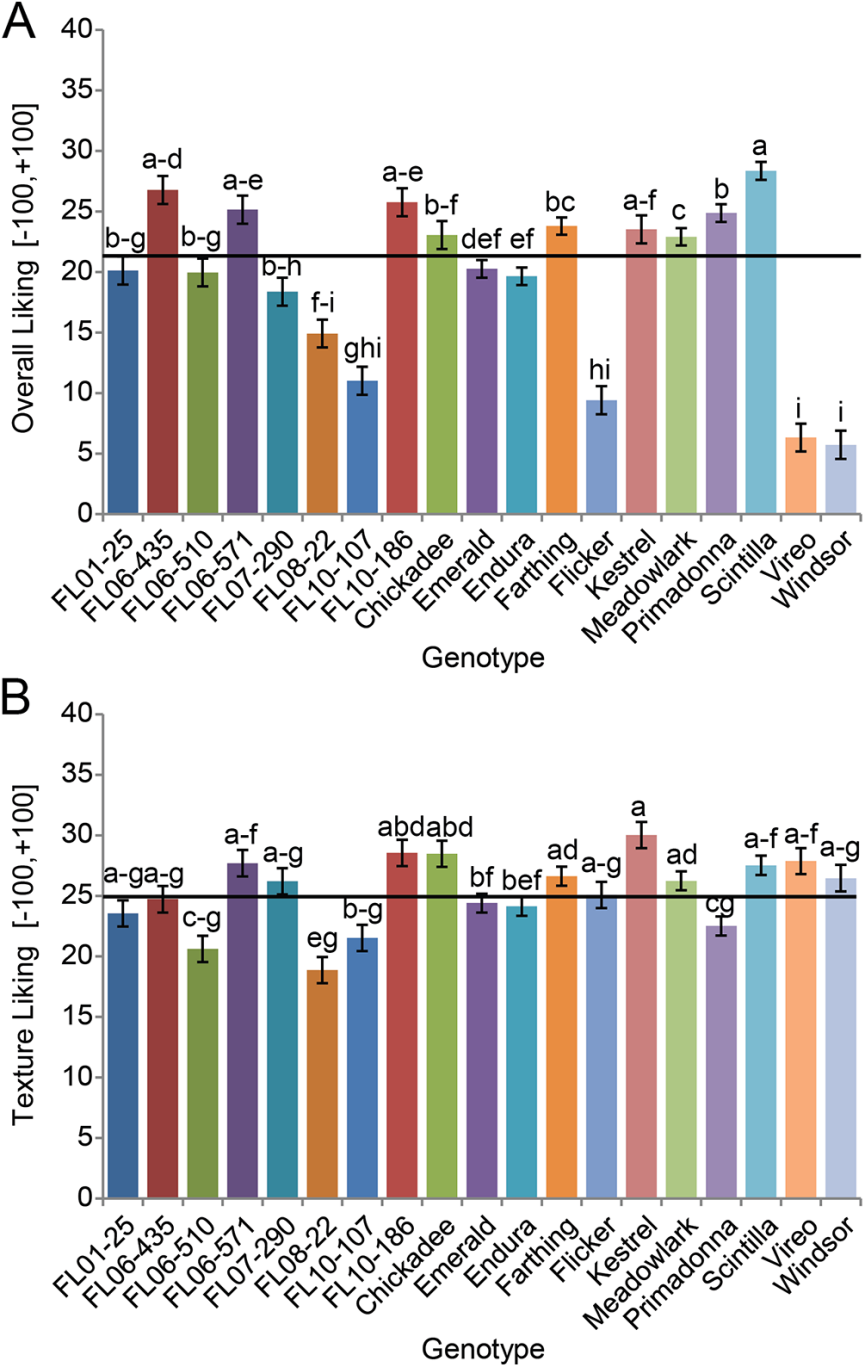
Hedonic ratings of blueberry genotypes. Mean ratings and standard error of 19 blueberry genotypes for (A) overall liking and (B) texture liking on a hedonic general Labeled Magnitude Scale (gLMS) (-100 to +100; -100 = greatest disliking of any kind, +100 = greatest liking of any kind). Overall mean is denoted by a black line. LSMeans were separated for responses of overall liking and texture liking with fixed effect of genotype and random effect of panelist using Tukey’s HSD (α = 0.05).

**Fig 3:**
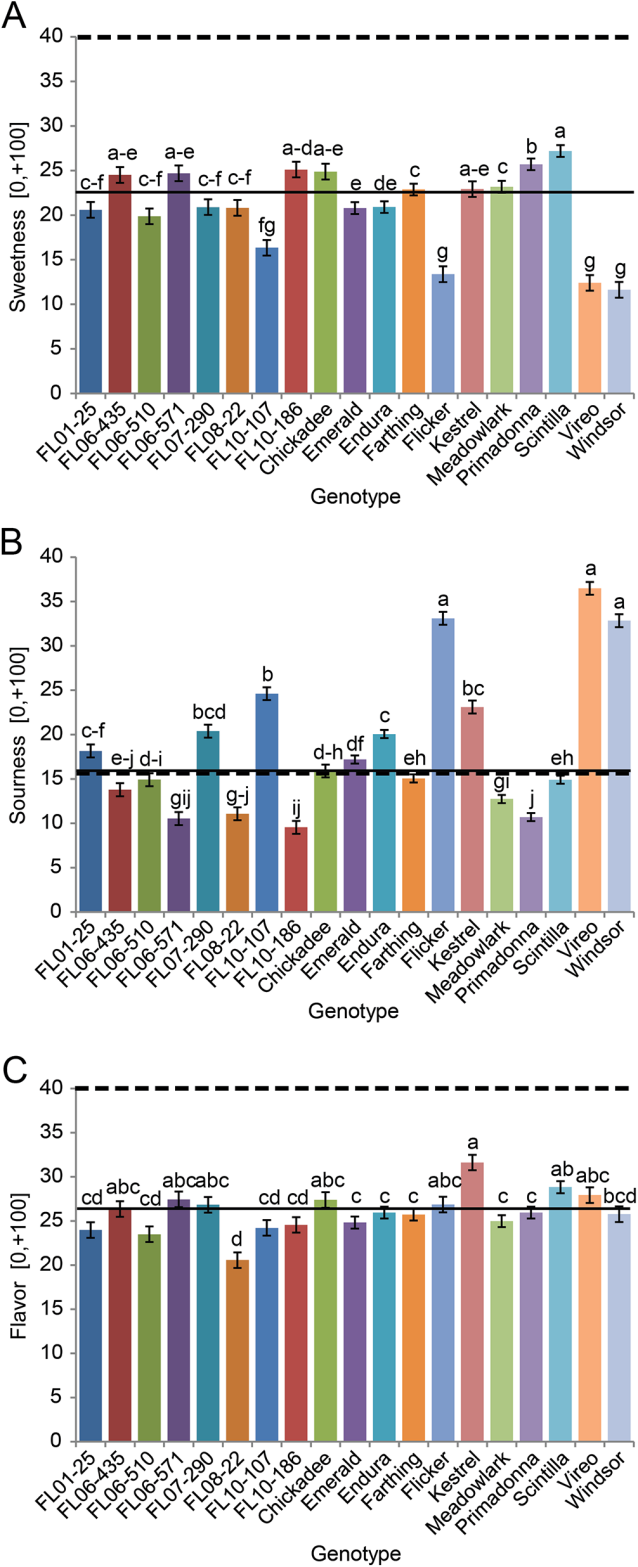
Sensory intensity ratings of blueberry genotypes. Mean ratings and standard error of 19 blueberry genotypes for (A) sweetness, (B) sourness, and (C) flavor on intensity general Labeled Magnitude Scales (0 to +100; 0 = no sensation, 100 = most intense sensation of any kind). Overall mean is denoted by a black line, mean ideal value by a dashed line. LSMeans were separated for intensity ratings of each sweetness, sourness, and flavor, with fixed effect of genotype and random effect of panelist using Tukey’s HSD (α = 0.05).

Of 153 blueberry samples assayed in sensory panels, every sample scored in the positive region of the hedonic scales (-100,+100) for overall liking and texture liking (S2 Table, Fig 2). Panelists scored berry texture on the hedonic gLMS scale to reduce artificially low scores in other scales in the incidence of poor texture. They then rated the individual sensory experiences of sweetness, sourness, and blueberry flavor on an intensity gLMS scale (0,+100) (Table 1, Fig 3). Additionally, panelists were asked to assign intensities of these sensory experiences with regard to an ideal blueberry. No genotype met panelists’ ideal sweetness intensity (Table 1, Fig 3A). In previous sensory studies with tomato (*Solanum lycopersicum* L.) and strawberry (*Fragaria × ananassa* Duchesne), panelists rated actual samples using the same scales and also indicated ideal sensory intensities for these fruits [22,23]. This hypothetical blueberry value fell between the ideal sweetness scores (intensity gLMS scale) given for strawberry (42) and tomato (33) [22,23]. This expectation that blueberry be sweeter than tomato is understandable given tomatoes’ savory role in culinary preparation. However, the expectation that a strawberry be sweeter than a blueberry is curious given their similar uses as fresh fruit and dessert, and blueberry’s typically higher sugar content compared to strawberry.

**Table 1:** Summary of consumer sensory ratings of blueberry samples.

Attribute	Scale	Mean	Minimum	Maximum	Fold Difference	Mean Ideal^z^
Overall Liking	[-100,+100]	22	6 (Windsor)	28 (Scintilla)	4.9	-
Texture Liking	[-100,+100]	25	19 (FL08-22)	30 (Kestrel)	1.6	-
Sweetness	[0,+100]	23	12 (Windsor)	27 (Scintilla)	2.3	39
Sourness	[0,+100]	16	10 (FL10-186)	36 (Vireo)	3.8	16
Flavor	[0,+100]	26	21 (FL08-22)	32 (Kestrel)	1.5	41

Summary of 30 sensory panels (n = 72 to 109, average = 92 respondents per panel) assaying 19 blueberry genotypes for overall liking, texture liking, sweetness, sourness, and flavor intensities using hedonic general Labeled Magnitude Scales (gLMS) (-100 to +100; -100 = greatest disliking of any kind, +100 = greatest liking of any kind) and intensity gLMS (0 to +100; 0 = no sensation, 100 = most intense sensation of any kind).

^z^Panelists were also asked to rate the intensity of taste and flavor sensations of a hypothetical ideal blueberry on this scale.

On average, sourness was the lowest intensity experienced, but exhibited the most variation between berries among the three sensory intensities rated (Table 1, Fig 3B). The sourest berry, ‘Vireo,’ was twice the ideal sourness (36). ‘Chickadee’ came closest to having the ideal intensity of sourness for blueberries according to this group of panelists, and was rated just above average in overall liking. The genotype with the highest overall liking score, ‘Scintilla,’ was close to the ideal indicated sourness intensity (15; S2 Table). The congruence with overall liking, measured sourness intensity, and ideal sourness intensity suggests that panelists are indeed able to numerically express ideal sourness intensity.

Intensity of blueberry flavor exhibited the least variation in sample scores compared to perceived sweetness and sourness (1.5-fold variation; Table 1), and like sweetness, also fell short of the panelists’ ideal flavor intensity (Table 1, Fig 3C). The lack of variation in flavor intensity among the 19 genotypes assessed may be due to poor panelist understanding of flavor (as opposed to taste), limited genetic diversity in flavor in the germplasm, or a combination of the two. Panel favorite ‘Scintilla’ had an above average flavor intensity (29; S2 Table), second only to ‘Kestrel’ (32), which was also highly rated by panelists (24). In comparison, the ideal flavor intensities desired for strawberry and tomato were higher (45) [22,23]. Given the quantities of volatiles reported in the literature for these three fruits via comparable collection methods and expressed in in ng gFW^-1^ h^-1^, blueberry emits fifteen times the volatiles compared to tomato, and about a third the volatiles emitted by strawberry [22,23]. While this might explain why the panelists would expect an ideal strawberry to be more flavorful, it draws into question why the flavor intensity of an ideal tomato be higher than an ideal blueberry.

The discrepancy between consumer ideals and actual products highlights the potential for breeding blueberry sensory attributes that exceed consumer expectations, ideally leading to increased repeat purchases, and expanded market potential for the crop. The perceivable sensory differences among blueberry genotypes indicate that the previously reported diversity in biochemical makeup [12–19] is sufficiently variable to extract meaningful information relating to the sensory experience.

### Overall liking of blueberries is strongly related to favorable texture, intensities of sweetness, flavor and sourness

**Fig 4:**
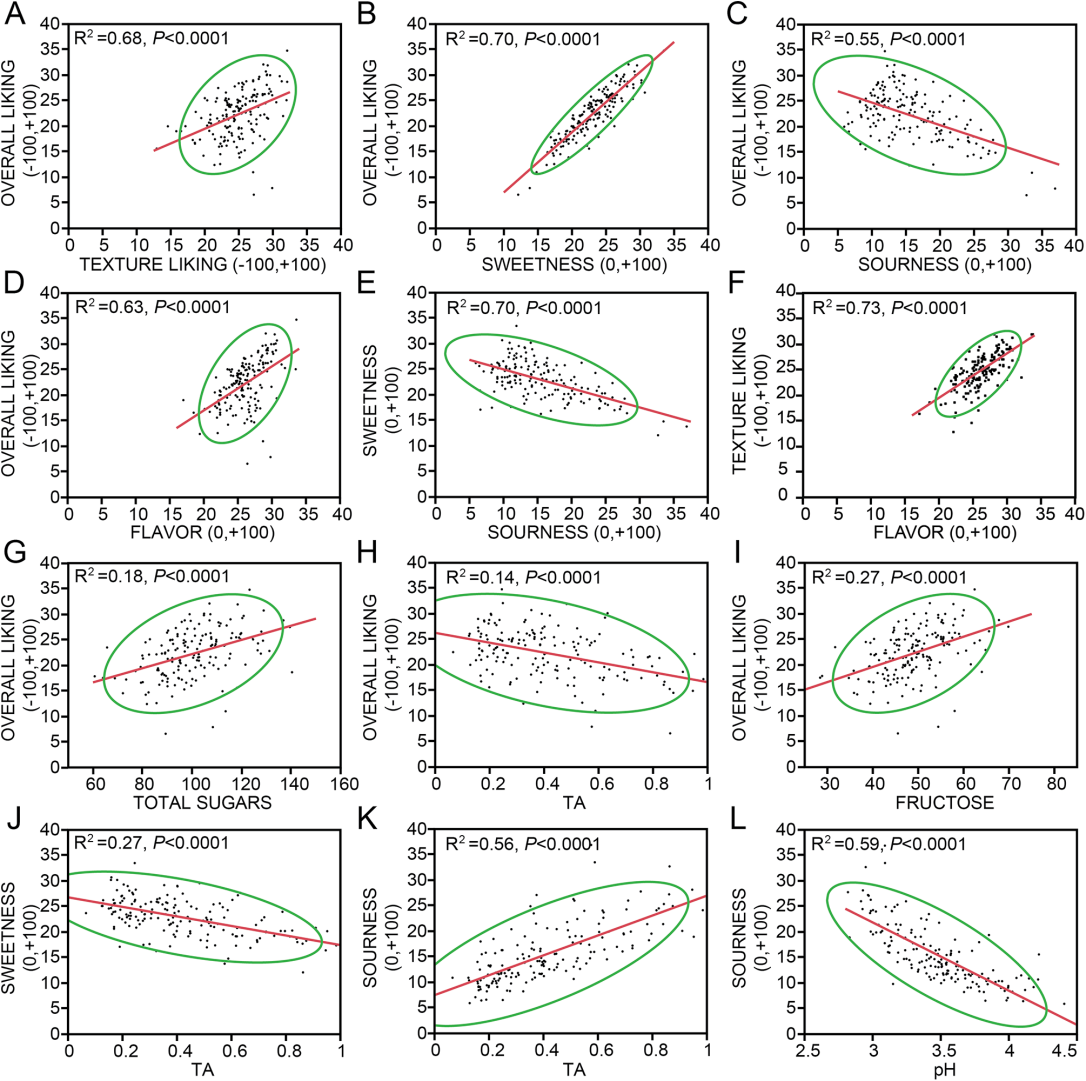
Significant pair-wise correlations between sensory measurements and primary biochemical components. Overall liking was fitted to (A) texture liking, (B) sweetness, (C) sourness, and (D) flavor. (E) Sweetness was fitted to sourness. (F) Texture liking was fitted to flavor. Raw sensory panel data was used with panelist treated as a random effect for A-F. The LSMeans of sensory responses per sample were fit to sample biochemical measures for G-L. Overall liking was also fitted to biochemical measures of (G) fructose and (H) TA. Sweetness was fit to biochemical measures of (I) fructose and (J) TA. Sourness was fitted to biochemical measures of (K) TA and (L) pH. Coefficient of determination (R^2^) and *P-*value of fit is listed with individual scatterplots. Line represents linear fit, and ellipse indicates 95% confidence range of data.

To determine which sensory predictors measured in this study influenced the overall blueberry eating experience, pair-wise correlations between each panelists’ scores of overall liking, favorability of texture, and the sensory intensity measures of sweetness, sourness, and flavor were individually fitted (S3 Table; Fig 4A–4F shown are select significant relationships). These four measures were all individually significant contributors to the response overall liking of blueberry fruit (*P*<0.001) with the best fit indicated by sweetness intensity (R^2^ = 0.70), followed by liking of berry texture (R^2^ = 0.68), and flavor intensity (R^2^ = 0.63). Sourness intensity had a significantly negative relationship with overall liking (R^2^ = 0.55). Overall liking increased as the texture was perceived as better, and as the fruit was more intensely sweet and flavorful. The relationship between overall liking and texture liking is a reasonable finding, as these are both rated on hedonic scales. If a panelist likes the berry, it is partially because they like that berry’s texture: the somatosensory experience of texture is inseparable from the rest of the sensory components [4]. That texture is such a strong sensory component of overall liking is at odds with our previously published blueberry perception study, in which flavor attributes were most indicative of purchase [3]. In the perception study, descriptions of poor blueberry texture, such as mealiness, seediness, and toughness, were the most powerful in decreasing blueberry purchase likelihood, but positive texture attributes (firmness, crispness) were generally perceived as neutral. This highlights a potential downside to consumer perception surveys: it is difficult to cognate the mechanical value of texture until it is experienced firsthand. Even then, texture is a complex experience that is difficult to parcel from other sensory components [4] which is the reasoning behind the use of the hedonic scale rather than some assemblage of descriptive intensity factors such as juiciness, firmness, and mealiness [24].

As the sourness of blueberry samples was perceived as more intense, the pleasure derived from the fruit decreased (Fig 4C). Blueberry sweetness (Y) and sourness (X) were negatively correlated with each other (R^2^ = 0.70, *P*<0.0001, Fig 4E) as expected due to mixture suppression of sweet and sour tastes [25,26]. When the panel means (as opposed to individual panelist scores) of sensory measures were fit against each other, fits for texture, sourness, and flavor against overall liking went down (R^2^ = 0.14, R^2^ = 0.35, R^2^ = 0.22, respectively, *P*<0.0001) while the fit for sweetness went up (R^2^ = 0.81, *P*<0.0001). Changes in fit may be due to the elimination of personal bias for these sensory parameters when averages were used rather than individuals’ associations between ratings. This may also be due to the large range of the gLMS scales, and the variation in range use of the scales among panelists, which is accounted for when each panelist can be treated as a random effect.

The relationship between flavor intensity (X) and texture liking (Y) was also linear (R^2^ = 0.73, *P*<0.0001, Fig 4F). It is common for interaction to occur between olfaction and somatosensation [4]. A significant relationship between blueberry flavor intensity and juiciness has been previously reported [27]. These correlations may be a result of optimally ripe samples, where texture is ideal and flavor compounds are at a maximum [15]. In a peach (*Prunus persica* L.) QTL mapping and fruit quality study, a locus controlling peach melting, firmness and several volatiles was found, suggesting either pleiotropic effects of a single gene upon these traits or an additional linkage locus [28]. However, the relationship between flavor and texture may be created post-fruit consumption. Differences have been found between whey protein gels of varying texture and their perceived flavor intensity, despite the fact that release of flavor compounds from the matrix was not changed due to gel texture differences [29], demonstrating the potential integration of these two discrete senses upon each other in the brain [4].

In considering primary biochemical drivers of the sensory experiences, it is known that sugars and acids are principal contributors to taste [30]. A commonly used indicator of fruit quality has been the ratio of soluble solids to titratable acidity of fruit. While SSC/TA was significantly correlated to overall liking, sweetness, and sourness in linear pairwise regressions, the individual measures SSC and TA explained more of the variance in sensory ratings than did the ratio (S3 Table). Overall liking was significantly positively correlated to concentrations of all sugars (best fit was Total Sugars, R^2^ = 0.20, *P*<0.001, Fig 4G) and negatively with increasing TA (R^2^ = 0.14, *P*<0.001, Fig 4H). Perceived sweetness is best explained by measures of sugars, including Fructose (R^2^ = 0.27, *P*<0.0001 Fig 4I), Total Sugars (R^2^ = 0.26, *P*<0.0001), SSC (R^2^ = 0.25, *P*<0.0001), and Glucose (R^2^ = 0.20, *P*<0.0001). Although still significant, measures of Sucrose are much less suitable as a predictor of sweetness (R^2^ = 0.08, *P* = 0.0004). As in previous reports [31,32], Sucrose was present in very low amounts in the blueberry fruit surveyed, accounting for 1.6% of total sugars on average (S2 Table). Sweetness was negatively correlated with TA (R^2^ = 0.27, *P*<0.0001, Fig 4J), while TA explained over half of perceived sourness (R^2^ = 0.56, *P*<0.0001, Fig 4K). An even better explanatory variable of perceived sourness was pH (R^2^ = 0.59, *P*<0.0001, Fig 4L). Of the top five best liked samples, pH measures ranged from 3.3 to 3.6, compared to the study range of 2.8 to 4.4 (S2 Table).

However, more sugar does not always equate to more sweetness. Of the top five sweetest samples, none of these is in the top five for total sugar concentration (S2 Table). In fact, ‘Primadonna’ (2014 H1—first harvest from Haines City in 2014) was the fourth sweetest berry among all thirty panels, and had a below average concentration total sugars. Conversely, ‘Emerald,’ (2013, H2), which had the highest total sugar concentration of all samples assayed (140.4 mg gFW^-1^ vs. average = 100.5 mg gFW^-1^), scored below average for sweetness intensity (21 vs. average 23) and liking (20 vs. average 22), underscoring the sensory importance of many other compounds in the fruit matrix. This ‘Emerald’ sample was very acidic (pH = 3.0, average = 3.5; TA = 0.91, average = 0.41), while the ‘Primadonna’ sample was sub-acid (pH = 3.6; TA = 0.18). Additionally, in both strawberry and tomato fruits, aromatic volatile compounds have been discovered to contribute to sweetness beyond the effects of sugars [22,23]. This introduces the possibility of breeding for a biochemical recipe that does not exclusively involve increasing sugar content and lowering acidity.

From these results, it is clear that panelists can discern genotypic variation in blueberry sensory components. However, delivering and marketing a blueberry product of premium quality, as defined by excellent texture, increased sweetness and flavor, and a balance of sourness, would necessitate identifying biochemical quality measures to benchmark specially marketed products such that they would consistently meet consumer expectations of a higher quality berry. This would only be realistic if certain genotypes could be identified where these biochemical components do not suffer from high variation due to environmental effects, thereby yielding reliable fruit quality.

### Relating the human experience to biochemical makeup

Since overall enjoyment of blueberries has been highly correlated with sweetness, flavor acceptability and blueberry-like flavor intensity (Fig 4) [14], and the sensory experience is influenced by numerous compounds released upon blueberry mastication, an appropriate model to detect complex connections between these disciplines was sought. In this study we used a PLS model to explain the hedonic and sensory components associated with eating blueberries using biochemical measurements [33–37]. Texture liking, for which no physical measure was made, was included in the explanatory variables, along with glucose, fructose, sucrose, TA, pH, and 52 volatile compounds, to explain the sensory components of overall liking, sweetness, sourness, and flavor. When texture liking was included, the model explained 70% of variation in the sensory measures. Upon removing texture liking from the model, 64% of the variation in sensory components was explained (Table 2). The 6% increase in variation explained accounted for by texture is expected by the large contribution of texture liking to overall liking and flavor of blueberry fruit (Fig 4A).

**Table 2:** Blueberry biochemical measures explaining hedonic and sensory intensity ratings.

Response	Percent Explained^z^	Factor	PLS Coefficient
**Overall Liking**	68%	Fructose	0.2159
		β-Caryophyllene oxide (CAS#1139-30-6)	0.1455
		Glucose	0.1433
		Neral (CAS#106-26-3)	0.1170
		pH	0.0907
		2-Heptanone (CAS#110-43-0)	0.0871
		Phenylacetaldehyde (CAS#122-78-1)	-0.0816
		Linalool (CAS#78-70-6)	-0.0836
		Hexanoic acid (CAS#142-62-1)	-0.0999
		E-2-Hexenal (CAS#6728-26-3)	-0.1211
		1,8-Cineole (CAS#470-82-6)	-0.1912
		TA	-0.2015
**Sweetness**	71%	Fructose	0.2237
		Glucose	0.1504
		β-Caryophyllene oxide (CAS#1139-30-6)	0.1418
		pH	0.1319
		Neral (CAS#106-26-3)	0.1197
		2-Heptanone (CAS#110-43-0)	0.0922
		Phenylacetaldehyde (CAS#122-78-1)	-0.0777
		Linalool (CAS#78-70-6)	-0.0787
		Hexanoic acid (CAS#142-62-1)	-0.1003
		E-2-Hexenal (CAS#6728-26-3)	-0.1253
		1,8-Cineole (CAS#470-82-6)	-0.1925
		TA	-0.2402
**Sourness**	78%	TA	0.3105
		1,8-Cineole (CAS#470-82-6)	0.1444
		E-2-Hexenal (CAS#6728-26-3)	0.1378
		2-Nonanone (CAS#821-55-6)	0.1145
		Hexanoic acid (CAS#142-62-1)	0.0953
		Z-2-penten-1-ol (CAS#1576-95-0)	0.0726
		6-Methyl-5-hepten-2-one (CAS#110-93-0)	-0.0666
		1-Hexanol (CAS#111-27-3)	-0.0783
		Fructose	-0.0863
		Methyl isovalerate (CAS#556-24-1)	-0.0925
		Neral (CAS#106-26-3)	-0.0929
		pH	-0.2573
**Flavor**	60%	Fructose	0.1856
		β-Caryophyllene oxide (CAS#1139-30-6)	0.1527
		2-Undecanone (CAS#112-12-9)	0.1404
		Glucose	0.1392
		2-Heptanone (CAS#110-43-0)	0.1304
		3-Methyl-1-butanol (CAS#123-51-3)	0.0960
		Phenylacetaldehyde (CAS#122-78-1)	-0.0625
		Methyl isovalerate (CAS#556-24-1)	-0.0625
		Linalool (CAS#78-70-6)	-0.0823
		Methyl hexanoate (CAS#106-70-7)	-0.0861
		1,8-Cineole (CAS#470-82-6)	-0.0876
		pH	-0.1264

Top six and bottom six biochemical measures explaining sensory responses as determined by partial least squares (PLS) analysis. Model explains 64% of the variation in sensory components of overall liking, sweetness, sourness, and flavor with biochemical measures of glucose, fructose, sucrose, TA, pH, and 52 volatile compounds.

^**z**^Percent variation of each individual sensory parameter explained by the biochemical measures in the overall model is presented

The variation of each sensory aspect explained by the six highest and lowest biochemical effectors is shown in Table 2. The sensory parameter with the most variation explained by biochemical measures was sourness, with 78% accounted for. The monosaccharide fructose was the most impactful effector of overall liking, sweetness and flavor, and detractor from sourness (Table 2). Its structural isomer glucose was also important in overall liking, sweetness, and flavor, but did not act to decrease sourness in this model. Fructose has been shown to be perceivably sweeter than glucose at equivalent concentrations [38–40]. It is known that the human sweetness receptor on the tongue undergoes varying conformational changes depending on the structure of the ligand that binds (e.g. fructose vs. glucose vs. artificial sweeteners), resulting in different degrees of perceived sweetness [41]. In the present study, sucrose did not contribute significantly to sweetness as it only accounts for approximately 1–2% of total blueberry sugars. Acidity as determined by pH also appeared to be important in all four sensory components assayed. TA was not associated with flavor but is a strong factor in the other three sensory components.

Flavor had a strong relationship with fructose and pH, in addition to several volatiles also implicated in contributing to sweetness and sourness in this dataset (Table 2). The influence of pH and fructose on the olfactory-mediated response of flavor may be due to difficultly for untrained consumer panelists to isolate flavor from taste. However, this could also be due to the integration of aromatics with taste in the fruit matrix and the brain; for example, pH can affect volatile emission in fruit tissue [42], and relative concentrations of compounds in solution can dramatically change their associated olfactory responses [43]. Interactions between taste and retronasal olfaction are also known to occur. For example, sweetness and other aromas have been shown to be enhanced depending on the combination of taste molecules and volatile molecules present in an ingested sample [6,22,23]. In the discussion of volatile compounds and their contribution to flavor, it should be noted that the specific aromatic descriptors of volatiles in the literature are limited in that concentration and interaction with the fruit matrix may alter sensory perception [43–45].

Some of the volatiles included in the PLS model contribute to the explanation of more than one sensory descriptor as rated by the panelists. Volatile compounds β-caryophyllene oxide (CAS#1139-30-6) and 2-heptanone (CAS#110-43-0) are linked to an increase in overall liking, sweetness, and flavor in blueberries. β-Caryophyllene oxide has been identified in black currant (*Ribes nigrum* L.), black ground pepper (*Piper nigrum* L.), and hops (*Humulus lupulus* L.) and has been described orthonasally as “spicy” [46–48]. ‘Meadowlark’ constitutes samples with the highest concentrations of 2-heptanone, distributed across all years (2012–2014) and all locations (UFPSREU, Haines City, Waldo). 2-Heptanone has also been reported in strawberry, apple (*Malus domestica* Borkh.), and passion fruit (*Passiflora edulis* Sims) [49–51].

Another pervasive compound, neral (CAS#106-26-3), is positively associated with liking and sweetness, but negatively associated with sourness. Neral is a geometric isomer of geranial, which together are recognized as ‘citral,’ and characteristic of lemon (*Citrus × limon* L.) [52]. Although geranial is also found in these blueberry samples, it was not identified as contributing to the sensory experience, highlighting the point that enantiomers may have significantly different sensory qualities.

The favorability of the fruit was negatively associated with several volatile compounds in this study, including two compounds that have often been associated with typical blueberry flavor in literature, linalool (CAS#78-70-6) and 1,8-cineole (CAS#470-82-6) [19, 53–55]. In this PLS model, linalool and 1,8-cineole detracted from overall liking, sweetness and flavor. 1,8-Cineole also contributed to sourness. 1,8-Cineole was highest in samples which were harvested in the earliest part of the season (late March). Interestingly, linalool was highest in samples which were all harvested from the Waldo location (high tunnel production). *E*-2-Hexenal (CAS#6728-26-3), which has been described orthonasally as fresh, leafy green, floral, sweet, and pungent [44], also reduced liking and sweetness in the model, and was a positive effector of sourness. This compound constituted the largest proportion of total volatile emission, and was especially high in early season selections during 2014.

Two volatiles that were unique to explaining increases in blueberry flavor intensity were 2-undecanone (CAS#112-12-9) and 3-methyl-1-butanol (CAS#123-51-3). 2-Undecanone was characteristic of the variety ‘Scintilla.’ It has been previously reported in red raspberries (*Rubus idaeus* L.) and blackberries (*Rubus* spp. *hyb*), and has been described orthonasally as floral and citrusy [56,57]. 3-Methyl-1-butanol–a sweet, fruity, brandy-like compound found in tequila (*Agave tequilana* Weber), and grape (*Vitis vinifera* L.) and apple wines–was produced in the largest quantities by ‘Farthing’ and ‘Scintilla’ [58–60]. Increasing these two volatile compounds in the breeding germplasm could result in increased flavor intensity, as desired by blueberry consumers.

This model could be used to target up- and downregulation of particular compounds in blueberries for increased consumer favor. To develop a better tasting berry based on this model, breeders may want to consider targeting pH between 3.2 and 3.5, increasing sugars, particularly fructose, β-caryophyllene oxide, 2-heptanone, neral, 2-undecanone, 3-methyl-1-butanol, and decreasing linalool, 1,8-cineole, and *E*-2-hexenal. However, also of consideration to the breeder is the reliability of biochemical production of these compounds in fruit, as determined by genetics, environment, and their interaction effects.

### G×E Effects

To further develop a realistic set of breeding targets for improved flavor, a G×E study was conducted to quantify the variation exhibited by these target compounds and to indicate which were most available to manipulate genetically. Six of the genotypes (‘Emerald,’ ‘Endura,’ ‘Farthing,’ ‘Meadowlark,’ ‘Primadonna,’ and ‘Scintilla’) used to sample biochemical and sensory variance were grown in three locations and harvested over the course of three years to determine the effects of genotype and environmental variables on taste and flavor compounds. Volatile emission has previously been shown to vary by location in strawberry, ginger (*Zingiber officinale* Roscoe), thyme (*Thymus daenensis* Celak and *Thymus vulgaris* L.), and peaches [28,61–64] and between open field and protected structures in strawberry, raspberry, and tomato [10,61,65]. Geographical and climactic differences of the locations in this study are listed in S4 Table. Blueberry plants grown at the Waldo location were grown under high tunnels while plants at UFPSREU and Haines City were open field.

To determine the source of variance for the biochemical effectors of flavor (Table 2), a linear model in which E was broken down into Location (L) and Year (Y) was constructed. The significance and percent variance for each term in the model are listed in Table 3. All compounds besides hexanoic acid were significant for genotypic variation (*P*<0.0001). In contrast, methyl hexanoate (CAS#106-70-7) and linalool exhibited only effects of G (*P*<0.01). 6-Methyl-5-hepten-2-one (CAS#110-93-0) had the highest amount of variation sourced from genetics (89%). 6-Methyl-5-hepten-2-one has been shown to be a conversion product from the terpenoid citral (isomeric mixture of neral and geranial) by citral lyase in *Penicillium digitatum* spores [66], and does indeed have a significant negative pair-wise relationship to both neral and geranial in this blueberry biochemical study (*P* = 0.03, *P*<0.0001, respectively). Other compounds that had large genetic influence were 2-undecanone (82%), 2-nonanone (CAS#821-55-6) (80%), linalool (70%), and 1,8-cineole (51%). Formation of 2-undecanone is mediated by 3-oxolaurate decarboxylase [67]. Like 2-undecanone, linalool and 1,8-cineole also come from the terpenoid pathway and are both preceded by substrate geranyl diphosphate [68]. The high degree of genetic control over these compounds makes them ideal targets for breeding for improved blueberry flavor, and knowledge of their enzymatic and genetic regulation opens the potential for marker development.

**Table 3:** Effects of genotype, year, and location on blueberry biochemical measures.

	*P* Values (Percent Variation)							
Compounds	L^z^	Y	L×Y	G	L×G	Y×G	G×L×Y	Total^y^
Fructose	0.012 (3)	0.054 (3)	0.017 (4)	**<0.0001** (31)	0.019 (8)	0.523 (3)	**0.009** (13)	65
Glucose	**<0.001** (6)	**<0.0001** (16)	**0.003** (5)	**<0.0001** (19)	0.086 (6)	0.427 (4)	0.172 (10)	65
pH	**<0.0001** (30)	**<0.0001** (6)	0.267 (0)	**<0.0001** (14)	0.017 (1)	0.574 (1)	0.052 (1)	52
TA	**<0.0001** (18)	**<0.0001** (13)	0.007 (5)	**<0.0001** (21)	0.192 (5)	0.435 (2)	**0.009** (11)	74
Methyl hexanoate (CAS#106-70-7)	0.045 (5)	0.023 (3)	0.154 (5)	**<0.0001** (22)	0.144 (5)	0.078 (8)	0.625 (6)	53
2-Heptanone (CAS#110-43-0)	**0.002** (3)	0.163 (1)	0.029 (4)	**<0.0001** (54)	**0.002** (7)	0.268 (2)	**0.003** (7)	79
6-Methyl-5-hepten-2-one (CAS#110-93-0)	**<0.001** (1)	0.633 (0)	0.542 (0)	**<0.0001** (89)	0.022 (1)	0.046 (1)	0.674 (1)	94
1-Hexanol (CAS#111-27-3)	0.018 (3)	**<0.0001** (46)	0.029 (3)	**<0.0001** (12)	**<0.001** (10)	0.137 (3)	0.659 (3)	79
2-Undecanone (CAS#112-12-9)	0.241 (0)	**0.001** (2)	0.023 (1)	**<0.0001** (82)	0.013 (2)	0.169 (1)	**0.003** (3)	91
β-Caryophyllene oxide (CAS#1139-30-6)	**<0.0001** (3)	**<0.0001** (4)	0.236 (1)	**<0.0001** (71)	**<0.001** (4)	0.020 (3)	0.029 (4)	89
3-Methyl-1-butanol (CAS#123-51-3)	**0.008** (5)	0.047 (7)	0.843 (2)	**<0.0001** (24)	0.196 (8)	0.429 (5)	0.92 (4)	54
Z-2-penten-1-ol (CAS#1576-95-0)	0.038 (4)	**<0.0001** (11)	0.034 (8)	**<0.0001** (18)	0.039 (10)	0.302 (5)	0.198 (8)	62
1,8-Cineole (CAS#470-82-6)	0.026 (2)	**0.004** (4)	**<0.001** (6)	**<0.0001** (51)	**<0.001** (8)	0.044 (2)	**<0.0001** (10)	84
Neral (CAS#106-26-3)	0.131 (3)	**0.004** (5)	**0.002** (9)	**<0.0001** (18)	**<0.001** (15)	0.030 (9)	0.151 (8)	66
Methyl isovalerate (CAS#556-24-1)	**0.005** (3)	0.324 (1)	0.608 (2)	**<0.0001** (43)	**<0.001** (11)	0.644 (2)	0.825 (4)	66
E-2-Hexenal (CAS#6728-26-3)	**0.001** (6)	**<0.0001** (20)	0.020 (3)	**<0.0001** (27)	0.147 (4)	0.004 (6)	0.002 (12)	77
Linalool (CAS#78-70-6)	0.280 (1)	0.062 (1)	0.135 (2)	**<0.0001** (70)	0.494 (2)	0.051 (3)	0.284 (4)	82
2-Nonanone (CAS#821-55-6)	0.032 (1)	**<0.0001** (2)	**0.006** (1)	**<0.0001** (80)	**<0.001** (3)	**0.005** (2)	**<0.001** (5)	93
Hexanoic acid (CAS#142-62-1)	0.068 (2)	**<0.0001** (14)	0.121 (3)	0.066 (7)	0.992 (1)	0.146 (9)	0.999 (3)	39
Phenylacetaldehyde (CAS#122-78-1)	0.964 (0)	0.170 (7)	0.027 (3)	**0.001** (11)	0.437 (2)	0.404 (10)	0.347 (3)	36

Partition of the variation of the top 20 blueberry biochemical measures due to effects of genotype (G), year (Y), and location (L), and all corresponding interactions. *P<*0.01 are bolded.

^z^Proportion of variance (in parentheses) explained of each model term of effects genotype, location, year, and all interactions.

^y^Total variation of the compound explained by the model was summed, remaining variation is attributed to residual effects.

Seven biochemical measures had significant G×L×Y interaction effects, including fructose and TA (Table 3). Glucose varied most based on berry genotype (19%) and year of harvest (16%). Fructose had a higher contribution of genetics at nearly a third. pH varied simply by location (30%) and year (6%) with no interaction effects. 1-Hexanol (CAS#111-27-3) and *E*-2-Hexenal were the compounds most variable from year to year. *E*-2-Hexenal has also been reported as having strong environmental effects in peach [28]. These compounds would make poor breeding targets due to their susceptibility to environmental variation. Although β-caryophyllene oxide exhibited L×G effects, 71% of its variation can be accounted for through genotypic effects alone (Table 3). This oxidized sesquiterpenoid is the main product of a terpene synthase, which produces a number of volatile sesquiterpenes. Badoc and Lamarti [69] indicated that tropical climates favor the formation of oxidized forms of volatiles in dill (*Anethum graveolens* L.), and in agreement, β-caryophyllene oxide concentrations increase as latitudes decrease in this study, although further studies would have to be conducted to confirm this correlation. 2-Heptanone also exhibited interaction effects of L, G, and Y, yet 54% of variation in its emission appears to be controlled genetically. Conversely, neral emission is less genetically controlled (18%) and is equally affected by L×G interaction effects (15%). These analyses suggest the potential to focus breeding resources on upregulating β-caryophyllene oxide and 2-heptanone rather than compounds such as neral.

After focusing on compound performance across locations and years, we determined the significant variability of compounds for each genotype (*P*<0.01) in the event that less predictable compounds could be prone to tighter biochemical regulation in a particular genetic background (Table 4). ‘Emerald’ and ‘Scintilla’ had significant variability in the least number of compounds. ‘Endura’ was significantly variable for the highest number of compounds. ‘Endura’ was the only genotype to significantly vary for fructose concentration. Glucose was not variable in ‘Farthing’ and ‘Scintilla.’ ‘Scintilla’ also did not vary for pH or TA. All other genotypes had significant genotypic effects on pH. Based on these results, ‘Scintilla’ would be a good individual to use in crosses aimed at incorporating genetically regulated flavor compounds into new progeny because compounds tend to be predictable regardless of year and location, coupled with high sensory scores for the cultivar.

**Table 4:** Blueberry biochemical variation by genotype.

	Genotypes					
Compounds	Endura	Emerald	Farthing	Meadowlark	Primadonna	Scintilla
Fructose	**0.003**	0.015	0.072	0.219	0.364	0.842
Glucose	**<0.0001**	**0.0005**	0.028	**0.0006**	**0.001**	0.176
pH	**<0.0001**	**0.0002**	**<0.0001**	**<0.0001**	**<0.0001**	0.062
TA	**0.0009**	**0.001**	**<0.0001**	0.054	0.010	0.077
Methyl hexanoate (CAS#106-70-7)	**0.007**	0.864	0.661	0.019	0.681	0.522
2-Heptanone (CAS#110-43-0)	0.222	0.237	**<0.0001**	**<0.0001**	0.152	**0.001**
6-Methyl-5-hepten-2-one (CAS#110-93-0)	0.928	0.950	0.984	**0.0002**	0.027	0.996
1-Hexanol (CAS#111-27-3)	**<0.0001**	0.858	**<0.0001**	**0.005**	**0.002**	0.593
2-Undecanone (CAS#112-12-9)	**<0.0001**	0.834	0.990	**0.0009**	0.992	**0.002**
β-Caryophyllene oxide (CAS#1139-30-6)	**<0.0001**	0.421	0.152	**<0.0001**	0.019	**0.001**
3-Methyl-1-butanol (CAS#123-51-3)	0.010	0.718	**0.007**	0.583	0.424	0.819
Z-2-penten-1-ol (CAS#1576-95-0)	0.148	0.329	**0.002**	0.102	**0.003**	0.449
1,8-Cineole (CAS#470-82-6)	**<0.0001**	0.191	**<0.0001**	0.762	0.082	0.788
Neral (CAS#106-26-3)	**0.002**	0.236	**0.0009**	0.830	**0.007**	0.202
Methyl isovalerate (CAS#556-24-1)	0.984	1.000	0.932	0.021	**<0.0001**	0.953
E-2-Hexenal (CAS#6728-26-3)	**0.0003**	0.269	**<0.0001**	0.013	**<0.0001**	**0.007**
Linalool (CAS#78-70-6)	0.298	**0.003**	0.885	0.085	0.266	0.964
2-Nonanone (CAS#821-55-6)	**<0.0001**	0.925	0.855	0.035	0.601	**<0.001**
Hexanoic acid (CAS#142-62-1)	1.000	0.751	0.333	0.013	**<0.0001**	0.332
Phenylacetaldehyde (CAS#122-78-1)	0.074	0.205	0.022	0.070	0.997	0.530
# *P*<0.01	12	4	9	7	8	5

Significance of variation (location and year) of the top 20 biochemical measurements that explain sensory responses in blueberry when partitioned by genotype. Significance levels *P*<0.01 are bolded.

## Conclusions

Genotypic variation exists for sensory components, and there is a large perceivable range of derived pleasure and intensity of sensory components when eating blueberries. The blueberry flavor formula we present may not be applicable to all humans due to genetic variation in human sensory perception: there are approximately 400 genes that encode olfactory receptors in humans, among which nearly 6000 polymorphic events have been identified, resulting in high allelic diversity between individuals [43,70]. However, by collecting sensory and biochemical data from multiple year, location, and genotype trials, we sought to generate a broad enough sample to develop a robust model for improving blueberry flavor based on breeding for specific biochemical compounds. By conducting this G×E analysis, we were able to identify certain biochemical compounds that were associated with increased or decreased consumer liking and had a low degree of environmental variation. These compounds have thus become the primary targets to improve blueberry flavor in our breeding program.

## Methods

### Ethics statement

The field studies reported here did not involve endangered or protected species. Two locations utilized for this research (Waldo, FL and Haines City, FL) were privately-owned agricultural farms. The owners of both locations provided permission to conduct the research trial. The third location (UF Plant Science Research and Education Unit) is the primary agricultural research site for the University of Florida and no permission is needed to conduct research for UF faculty. Consumer panels were conducted at the Food Science and Human Nutrition Department at the University of Florida (UF) in Gainesville, FL. The UF Institutional Review Board 2 (IRB2) chaired by Ira S. Fischler approved the protocol and written consent form (case #2003-U-0491) which participants are required to complete.

### Plant material and environmental data

Southern highbush blueberry (*V*. *corymbosum* L.) cultivars and advanced breeding selections evaluated in this study were ‘Chickadee™’ (‘FL04-235’), ‘Emerald,’ ‘Endura™’ (‘FL06-377’), ‘Farthing,’ ‘Kestrel™’ (‘FL02-40’), ‘Flicker™’ (‘FL96-43’), ‘Meadowlark™’ (‘FL01-173’), ‘Primadonna,’ ‘Scintilla,’ ‘Vireo™’ (‘FL05-107’), ‘Windsor,’ ‘FL01-25,’ ‘FL06-435,’ ‘FL06-510,’ ‘FL06-571,’ ‘FL07-290,’ ‘FL08-22,’ ‘FL10-107,’ and ‘FL10-186.’ Some recent cultivar releases indicated above have utilized the selection testing number for plant patent protection while a trademarked name is recognized in commerce. The trademarked names will be used throughout this manuscript (‘Chickadee,’ ‘Endura,’ ‘Kestrel,’ ‘Flicker,’ ‘Meadowlark,’ and ‘Vireo’). Relatedness of all genotypes based on pedigree information is depicted in Fig 1A. Three kg of fruit was hand-harvested from either the UF Plant Science Research and Education Unit (UFPSREU lat. 29°24'37''N, long. 82°10'12' W) or two grower-cooperator farms located near Waldo, FL (lat. 29°46'8''N, long. 82°7'53' W), and Haines City, FL (lat. 28°3'26''N, long. 81°33'50''W) the day prior to the consumer sensory panel and stored overnight at 4°C. 200 g of fruit was retained for biochemical analyses.

Genotypes investigated for G×E analysis were ‘Emerald,’ ‘Endura,’ ‘Farthing,’ ‘Meadowlark,’ ‘Primadonna,’ and ‘Scintilla.’ These six genotypes were grown in two locations (UFPSREU and Haines City, FL) and five genotypes were grown in a third location (Waldo, FL). Blueberry plants at UFPSREU and Haines City were grown under open field conditions while plants at Waldo were grown under protective high tunnels.

Weather stations were posted at the three sites, which recorded temperature, relative humidity, and rainfall. Solar radiation measures were obtained from the Florida Automated Weather Network (FAWN) stations at or closest to each site (UFPSREU FAWN, Putnam Hall FAWN for Waldo, Lake Alfred FAWN for Haines City).

### Sensory analysis

Over the course of three years, 19 blueberry genotypes were evaluated in 30 sensory panels by 217 panelists, with an average of 92 panelists per panel. Panelists were trained with the scaling methods and were encouraged to return for the remainder of the study. Panelists rated overall liking, texture liking, sweetness, sourness, and flavor intensity using hedonic general Labeled Magnitude Scales (gLMS) (-100 to +100; -100 = greatest disliking of any kind, +100 = greatest liking of any kind) and intensity gLMS (0 to +100; 0 = no sensation, 100 = most intense sensation of any kind), which allow for improved comparison between panelists and between years [71,72]. Panelists were also asked to rate the intensity of taste and flavor sensations of a hypothetical ideal blueberry on this scale. Each panelist received two to four berries of five to six genotypes in a single panel in a Williams design, which was generated by Compusense 5.6 (Compusense Inc., Guelph, Ontario, Canada). They were asked to eat the blueberries of each genotype at once, and to take a bite of cracker and sip of water in between samples. Ratings were recorded using Compusense software.

### Measurement of biochemical components

Soluble solids content (SSC), titratable acidity (TA), and volatiles were quantified as in Gilbert *et al*. [3]. The volatiles were collected for two hours via dynamic headspace with HaySep Q 80–100 porous polymer adsorbent (Valco Instruments Company Inc., Houston, TX) and eluted with methylene chloride and nonyl acetate as an elution standard. Elutions were run on an Agilent 7890A Gas Chromatograph Flame Ionization Detector (GC-FID) DB-5 column (30 m length × 250 mm diameter × 1 mm film) for quantification and on an Agilent 7890A Gas Chromatograph coupled to an Agilent 5977A Mass Selective Detector (GC/MSD) for qualification (Agilent, Santa Clara, CA). Integrated peak area of each signal was normalized for elution, fitted to relative pure chemical response, corrected for sample mass, and expressed as nanograms per gram fresh weight per hour (ng gFW^-1^ h^-1^). Response fitting was calculated using the response factor of pure chemical standards (Sigma-Aldrich, St. Louis, MO) run on the same instrument (S1 Table).

Glucose, fructose, and sucrose were extracted via solid phase extraction (SPE) (adapted from [73]) and quantified on an Agilent 1260 Infinity Binary Liquid Chromatograph (LC) system coupled to an Agilent 6430 Triple Quadrupole (QQQ) (Agilent, Santa Clara, CA). Tissue was ground in N_2(l)_, 100 mg was dissolved in 1.9 mL buffer (74% HPLC-grade water, 20% methanol, 5% acetic acid, and 1% 0.15 M lactulose internal standard), spun down for 20 min, and the resulting supernatant was filtered using Captiva Premium syringe layered 0.45 μm filters (Agilent, Santa Clara, CA), evaporated to 1 mL in a vacuum centrifuge (Heto VR-maxi, Heto-Holten A/S, Allerød, Denmark) at 50°C, and then added to 4 mL acetonitrile. Mega BondElut NH2 columns (Agilent, Santa Clara, CA) were activated with 2 mL methanol followed by 5 mL 4:1 acetonitrile-water. The sample was then passed through the NH2 cartridge and sugars were eluted with 3 mL HPLC-grade water in an ultracentrifuge (Avanti J-25 Centrifuge, Beckman Coulter Inc., Brea, CA) at 5000 rpm for 5 min. Samples were diluted 25-fold to be run on the LC. Isocratic mobile phase flow rate of 0.4 mL min^-1^ consisted of 80% channel A, aqueous 0.1% formic acid, and 20% channel B, 0.1% formic acid in acetonitrile. The Agilent Hi-Plex H column (300 × 6.5 mm) was held at a constant temperature of 30°C. An injection volume of 10 μL was used for all biological, standard, and quality control samples. Electrospray ionization helium gas temperature was 350°C with a flow of 7 L min^-1^. Nebulizer pressure was at 60 psi and capillary potential was 2000 V. The QQQ was run in positive mode with an electron multiplier value of 200 and targets were observed using multiple reaction monitoring. A multiple compound standard series was used to establish individual compound response of sucrose, glucose, and fructose using MassHunter Quantitative Analysis (Agilent, Santa Clara, CA) software. Standards had a fixed lactulose concentration of 40 μM. Integrated area of sugars in biological samples were fitted to relative response, normalized for dilution, corrected for sample mass, and expressed as milligrams per gram fresh weight (mg gFW^-1^).

### Statistical analyses

#### Genotype, sensory and biochemical relationships

Genotypic relatedness based on pedigree and biochemical profiles were compared using hierarchical cluster analyses. Two-way ward cluster analysis of genotypic relatedness based on pedigree information was performed in R (R, Vienna, Austria). Two-way ward cluster analysis of genotypic relatedness based on biochemical profiles was constructed in JMP® Pro 10 (SAS Institute Inc., Cary, NC).

Pair-wise correlations and significance between sensory measurements and primary biochemical components were calculated, with panelist as a random effect. Least Square Means (LSMeans) of the sensory data for each sample were obtained to account of the random effect of panelist. A partial least squares (PLS) analysis was constructed in JMP® Pro 10 (SAS Institute Inc., Cary, NC) to reveal effectors of sensory ratings [33–37]. The response variables (Y) considered corresponded to LSMeans of overall liking, sweetness, sourness, and blueberry flavor intensity. Glucose, fructose, sucrose, TA, pH, and 52 volatile compounds (S2 Table) were considered as explanatory variables (X). The inclusion of texture liking was evaluated independently given that no explanatory physical measure was made for sample texture. Variables were centered and scaled (hence, correlation matrices were used), and a 20-fold cross-validation method was used. The model was created without texture for analysis.

#### Partitioning biochemical variation due to genetics and environment

A partition of the variation was done by fitting a linear model to each biochemical compound based on the three year G×E study using the genotypes were ‘Emerald,’ ‘Endura,’ ‘Farthing,’ ‘Meadowlark,’ ‘Primadonna,’ and ‘Scintilla’ that were grown in three sites and harvested three times per year for three years, with the exception of ‘Scintilla,’ which was not grown at Waldo:

$$y_{ijk}=\mu +G_{i}+L_{j}+Y_{k}+{\left(G\times L\right)}_{ij}+{\left(L\times Y\right)}_{jk}+{\left(G\times Y\right)}_{ik}+{\left(G\times L\times Y\right)}_{ijk}+\varepsilon_{ijk}$$

where *y*_*ijk*_ is the response of the *i*th genotype, in the *j*th location for the *k*th year, *μ* is the overall mean. *G*_*i*_ is the fixed effect of genotype, *L*_*j*_ is the fixed effect of location and *Y*_*k*_ is the fixed effect of year. All other terms correspond to interactions between the main factors. Environment was defined as the location within a given year. The residual term *ε*_*ij*_ was assumed to be normally distributed where all observations within the same environment were correlated based on a compound symmetry error structure. Significance of each of the model terms was done using an approximated F-test with the Kenward and Roger [74] correction for the degrees of freedom. The Studentized residuals of each variable were checked for normality before proceeding with the analysis. The specific genotypes levels were compared amongst each other and LSMeans were estimated. The proportion of variance explained of each model term was also obtained based on the Type III Sum of Squares. The model was fitted using PROC MIXED in SAS 9.4 (SAS Institute Inc., Cary, NC, USA).

## Supporting Information

S1 Table Chemical standards and response factors. (XLSX)

S2 Table Sensory ratings and biochemical measures of all samples from 2012–2014. (XLSX)

S3 Table Pairwise relationships between sensory ratings and biochemical measures. (XLSX)

S4 Table Different environment characteristics (per location per season). (XLSX)
